# Unlocking the Potential of Cellular Guidance in Endodontics: Advancing the Process of Pulp Regeneration and Beyond

**DOI:** 10.7759/cureus.51651

**Published:** 2024-01-04

**Authors:** Tariq M Alharbi, Arwa M Thabet, Shaqran H Alabbadi, Majed Y Alhazmi, Hanan F Khan, Manar A AlRasheed, Noor A Al-Twalbeh, Abdulmalik S Alsuhaim, Nadeen S Alqahtani

**Affiliations:** 1 Endodontics, King Fahad General Hospital, Medina, SAU; 2 Endodontics, Speciality Dental Center, Medina, SAU; 3 Dentistry, Ministry of Health, Riyadh, SAU; 4 Dentistry, Umm Al-Qura University, Makkah, SAU; 5 Dentistry, Jazan University, Jazan, SAU; 6 Dentistry, Dar Al Uloom University, Riyadh, SAU; 7 Dentistry, Prince Mohammed Bin Abdulaziz Hospital, Madinah, SAU

**Keywords:** endodontic management, stem cell, endodontic treatment outcomes, endodontic regeneration, endodontic treatment

## Abstract

Regenerative endodontics represents a paradigm shift in dental therapy, with the potential to not only restore damaged dental tissues but also to preserve the vitality of teeth. At the heart of this innovative approach is cell homing, a technique that harnesses the body's own healing mechanisms by recruiting endogenous stem cells to the site of dental injury for effective tissue regeneration. This review delves into the intricate processes of cell homing in the context of regenerative endodontics, particularly focusing on its application in immature teeth with open apices. It examines the role of bioactive molecules, scaffolds, and growth factors in orchestrating cell migration and differentiation within the root canal space. In addition, the review addresses the current limitations in clinical practice, such as the challenges in completely regenerating the pulp-dentin complex and the unpredictability in long-term outcomes. It also explores future possibilities, including the potential for more refined and effective regenerative strategies. By providing a comprehensive overview of the current state of cell homing in regenerative endodontics, this article aims to contribute to the ongoing development of advanced therapeutic techniques that could revolutionize endodontic treatment and improve patient care.

## Introduction and background

Dental caries is the most commonly occurring dental disease worldwide, with the global prevalence estimated at 46.2% and 53.8% [1]. The clinical intervention for superficial carious lesions involves the excavation of caries and tooth restoration. Carious lesions may extend to the dentin and lead to reversible pulpitis; in such cases, vital pulp therapy, such as pulp capping, can prevent the lesion from spreading. However, in extreme situations, caries may extend beyond the dentin to the pulp and cause infection and irreversible pulpitis. Extensive endodontic treatment is then required, and it involves complete pulpectomy - the extirpation of both coronal and radicular pulp - followed by obturation with gutta-percha and sealers. However, this intervention has several long-term disadvantages. Endodontic treatment can cause the teeth to become brittle, and there is a risk of reinfection if the biomechanical preparation is not performed properly. There is also a tendency for periapical infection to develop due to microleakage from the crown. If the tooth fractures following endodontic treatment, the prognosis worsens and often leads to extraction. These drawbacks reflect the importance of vital pulp tissue in maintaining a tooth's health. Vital pulp tissue confers protection from infection, assists in the formation of reparative dentin, and contributes to the tooth's tensile strength [2].

Currently, several research efforts have focused on regenerative endodontics. This field of endodontics involves tissue regeneration using various agents, particularly in vital pulp therapy, to regenerate or revitalize the pulp-dentin complex [3]. The agents work by forming a dentin bridge that covers the exposed pulp with odontoblast-like cells and scar tissue, maintaining the pulp's vitality. However, several factors affect the successful outcome of this treatment, including the location of the injury, the age of the tooth, the material used for vital pulp therapy, and the integrity of the restoration [4]. Overall success rates for the endodontic regeneration procedures ranged from 50% to 98%, and the survival rates were between 94% and 100% [5].

Given the high prevalence of pulp necrosis and infection caused by dental caries, there is an urgent need to regenerate pulp-like tissue in the empty and disinfected root canal space. This will maintain the vitality and sensitivity of the tooth and lead to a good prognosis. An effective approach to regenerate pulp tissue in such cases is through the process of cell homing [6].

From a clinical perspective, cell homing could be a more viable treatment option, as it is easier to perform and more economical. Cell homing involves the active recruitment of endogenous cells, including mesenchymal stem/progenitor cells (MSCs), into an anatomical niche area [7-8]. The cell homing mechanism regenerates vital pulp tissue through the chemotaxis of mesenchymal progenitor or stem cells via biological signaling molecules. These MSCs can be recruited to the niche site either by intravenous injection or by disinfecting the root canal with antibiotics and calcium hydroxide. The latter is followed by the induction of bleeding from the periapical area, which leads to the formation of a blood clot (BC). This BC acts as a scaffold and induces the migration, proliferation, and differentiation of progenitor stem cells from the tooth's apex. These stem cells include stem cells from the apical papilla (SCAP), dental pulp stem cells (DPSCs), and bone marrow stem cells (BMSCs) [9-10]. The cell homing technique can be performed on immature teeth with necrotic pulps and open apices, as well as on mature teeth with necrotic pulps and narrow apices. However, some histological studies have found that cell homing led to the regeneration of cementum, bone, and periodontium-like tissues, instead of actual pulp tissue [11]. The pulp tissue regenerated from cell homing should have an architecture similar to that of natural pulp, including cellular and extracellular structure, vascularity, and innervation [12].

The objective of this literature review is to comprehensively examine the latest advancements in the clinical application of cell homing in the field of regenerative endodontics, with a specific focus on its use in immature teeth with open apices. In the following sections, this review will delve into the intricate mechanisms of cell homing, evaluate its efficacy and limitations in restoring vital pulp in challenging clinical scenarios, and discuss future possibilities for this promising field. By analyzing previous research studies and their clinical outcomes, we aim to shed light on the rapidly developing landscape of regenerative endodontic therapies and their roles in improving dental health care.

## Review

Pulp tissue

The pulp tissue is a highly vascularized and innervated tissue in the tooth. It consists of various types of cells, such as interstitial fibroblasts, endothelial cells, pericytes, and mesenchymal stem cells (MSCs)/progenitor cells, which are all held together in fibrous connective tissue. The main functions of the pulp are to produce, protect, and repair dentin, provide nourishment to the surrounding mineralized connective tissue, and impart sensitivity to the tooth. The pulp is organized into three different zones: the odontoblastic zone, the cell-free zone of Weil, and the cell-rich zone. The odontoblastic zone consists of a layer of odontoblasts underlying the surrounding dentin. These cells are responsible for dentin formation and extend outward to infiltrate the dentin; collectively, they are referred to as the dentin complex. The primary function of this odontoblastic layer is to form dentin, and this is a lifelong process. In the event of dentin damage, these cells also perform repair and restoration functions. If this layer is damaged, new odontoblast-like cells are recruited from deeper parts of the pulp. These cells originate from undifferentiated mesenchymal/progenitor cells, fibroblasts, pericytes,or dental pulp stem cells (DPSCs) [13]. The dentin formed by these cells differs slightly from the original dentin: it has fewer tubules and primarily serves to protect the pulp from external noxious stimuli [14].

The cell-free zone of Weil, which is located below the odontoblastic zone, contains the nerve plexus of Raschkow. This nerve plexus comprises large myelinated A-delta fibers and small unmyelinated C fibers. A-delta fibers cause rapid and sharp pain, while C fibers initiate slow and dull pain. This pain results from hydrodynamic forces in the dentinal tubules stimulating nerve endings [15]. The inner cell-rich zone comprises fibroblasts, undifferentiated mesenchymal cells, peripheral T cells, a few macrophages, granulocytes, mast cells, and plasma cells, packed in dense connective tissue that is highly vascularized and innervated. Even though pulp tissue is tiny and does not have any collateral blood supply, it has very high levels of blood flow at 40-50 ml/min/100 g [16]. This makes the pulp vulnerable to even the slightest stimuli, and repeated stimuli over a long period can induce pulpal damage, hypoxia, and necrosis in extreme situations [17]. Once the pulpal tissue is damaged beyond repair, the treatment involves the permanent removal of the pulp. However, this could leave the tooth brittle and susceptible to fracture, among other complications [18]. This underscores the importance of maintaining vital pulp tissue, which is necessary for the continued formation of roots and dentin wall thickening.

Ideally, the pulp regeneration procedure should not only replicate the structure of natural dental pulp but also restore its normal functions [12]. These functions include depositing new dentin, maintaining cellular density, and reestablishing vascular and nerve structures similar to those of the natural dental pulp [12]. The number of nerve fibers in the regenerated pulp determines the degree of sensory function restoration. Therefore, an indispensable part of endodontic tissue regeneration is the regeneration of normal innervation and vascularity. This can be achieved through the formation of pulp-like tissue in the pulp-dentin complex, which is innervated with both sympathetic and parasympathetic nerve fibers and consists of interstitial fibroblasts and progenitor cells capable of replenishing the pulp cells [19]. The new treatment regime of pulp regeneration via cell homing can reinstate tooth vitality and sensitivity, securing further growth.

In the field of endodontics, terms like “revascularization,” “regeneration,” “revitalization,” and “repair” are often used interchangeably, but each has its unique definition and significance. Revascularization specifically pertains to the restoration of blood flow in the pulp space of immature teeth following traumatic injuries that disrupt the original blood supply [20]. Regeneration refers to the process of recreating damaged tissue with a new tissue of a similar type, thereby reinstating its biological functions [21]. Revitalization goes beyond the mere reintroduction of blood vessels; instead, it refers to the renewal of non-specific vital tissue within the pulp [22]. Repair, distinct from the other terms, involves replacing damaged tissue with a different type of tissue that is typically unable to emulate the original functions of the pulp [23]. Collectively, these procedures fall under the umbrella of regenerative endodontic procedures (REPs), which denote various methods aimed at achieving organized repair of the dentin-pulp complex [24].

When discussing the desired features of the regenerated pulp, it is essential to consider the histological, morphological, and functional characteristics of the original pulp tissue. Typically, normal pulp comprises vessels, nerves, and odontoblasts lining the predentin. These structures provide nutrition to the dentin, enable response to external stimuli, and form the reparative dentin [2-3]. To successfully mirror these attributes, a regenerated pulp is expected to possess a similar cellular structure and density, the ability to form reparative dentin, sufficient vascularization, and adequate innervation to respond positively to pulp vitality testing.

During dentinogenesis, the regenerated pulp should ideally form reparative dentin [2-4]. However, research has shown instances where excessive mineralized tissue, such as cementoid or bone-like tissue, forms instead. These types of mineralized tissues tend to obliterate root canals, leading to a loss of vitality and complicating any further retreatment [25].

Another crucial aspect of regenerated pulp is revascularization, which is achievable only when there is effective communication between the pulp canal and the apical bone marrow, ensuring a proper blood supply [26]. This blood flow is vital for providing nourishment to both the surrounding tissue and the dentin [10].

Lastly, the importance of reinnervation is highlighted in the pulp’s capacity to respond to various stimuli, including thermal sensitivity and pain in response to inflammation and infection. Should a regenerated pulp fail to react to such stimuli, akin to a tooth treated with pulpectomy, the patient may not notice the onset of infection, and this could negatively impact future prognoses.

Regenerative endodontics via cell homing

The concept of cell homing centers on the repair or regeneration of pulp tissue through the chemotaxis of endogenous host cells, facilitated by biological signaling molecules. Accordingly, hedgehog signalling and cytokines are the most critical signaling molecules involved in pulp regeneration. They play a pivotal role in mobilizing progenitor cells and regulating their proliferation and differentiation [27]. There are two distinct cellular processes integral to cell homing: recruitment and differentiation. Recruitment refers to the directional migration of stem cells to the site of tissue damage, while differentiation is the process through which stem cells transform into mature cells. Essentially, the damaged pulp-dentin complex needs stem cells to differentiate into odontoblasts, neural cells, endothelial cells, and other cells involved in vessel formation [10,27].

Biological and histological basis of cell homing

Dental Stem Cells

Dental stem cells, crucial for cell homing techniques, are mesenchymal in origin and include various types, such as DPSCs, stem cells from human exfoliated deciduous teeth (SHEDs), stem cells from the apical papilla (SCAPs), periodontal ligament stem cells (PDLSCs), dental follicle stem cells (DFSCs), inflammatory periapical progenitor cells (iPACs), and bone marrow stem cells (BMSCs). DPSCs and SHEDs are frequently utilized in cell homing. DFSCs, discovered by Gronthos et al. in 2000 [28], have the ability to differentiate into multiple cell types. A significant study demonstrated that mobilized DPSCs in pulpectomized teeth led to pulp tissue regeneration [29]. These cells are preferred for tissue regeneration due to their multipotency, ease of isolation, and therapeutic potential. SCAPs, known for their high proliferative capacity and ability to differentiate without exogenous growth factors, are effective in cell homing, especially noted for less telomere shortening [26]. Even in the presence of periapical infection or apical periodontitis, SCAPs can differentiate into bone, vessels, and nerves, contributing to pulp regeneration [27]. In the case of immature teeth, SCAPs are primarily recruited from the apical papilla for regeneration purposes [26].

Growth Factors

Growth factors are essentially proteins or polypeptides that initiate a wide range of cellular activities when bound, such as migration, proliferation, differentiation, and maturation. They can be either endogenous or exogenous [30]. Endogenous growth factors are located in dentinal walls. Their release is triggered by various circumstances including exposure to bacterial acid, irrigation with sodium hypochlorite, ethylenediaminetetraacetic acid (EDTA), or stimulation with CaOH, mineral trioxide aggregate (MTA), and biodentin. These growth factors play a crucial role in the recruitment, differentiation, proliferation, and maturation of progenitor cells, which are essential for tissue regeneration in cell homing. There are many different types of growth factors, but the most relevant ones are described below [31].

Bone morphogenetic protein (BMP) is primarily sourced from the bone matrix and contributes to the synthesis of mineral matrix by stem cells. Platelet-derived growth factor (PDGF) originates from platelets, endothelial cells, and the placenta, and it facilitates stem cell proliferation. Transforming growth factor α (TGF-α) is found in macrophages. It is instrumental in the development of epithelial tissues and their structural formations [30-31]. Transforming growth factor β (TGF-β) is present in the dentin matrix, activated TH1 cells, and natural killer (NK) cells. It functions as an anti-inflammatory agent, promotes wound healing, and inhibits macrophage and lymphocyte proliferation. Nerve growth factor (NGF) is a protein secreted by neuronal tissues, and it supports the growth and survival of nerve cells. Fibroblast growth factor (FGF) is widely present in various cells and encourages an increase in stem cell count across different tissues.

There is evidence supporting the use of both exogenous and autologous growth factors in cell homing. In a previous study, hydrogel scaffolds impregnated with basic fibroblast growth factor (bFGF) were administered into the teeth with necrotic pulp, and apical healing and continued root development were observed [32]. Another method is to obtain these growth factors from the recipient’s blood. When injected into the root canal, they enhance regeneration. An in vivo study on immature beagle dogs showed enhanced migration, proliferation, and differentiation of stem cells after a growth concentrate was injected. Histological and immunohistochemical analyses confirmed the regeneration of the pulp-dentin complex after eight weeks [33].

Scaffolds or Biomaterials

A scaffold plays a crucial role in cell homing, serving as a three-dimensional structure with properties akin to the natural extracellular matrix. Essential requirements for a scaffold in cell homing include facilitating cell location; possessing sufficient physical and mechanical strength [34]; enabling the transport of nutrients, oxygen, and growth factors for cell proliferation and differentiation; and being inert, non-reactive, and biodegradable [35].

In cell homing, there are two main types of scaffolds: natural matrices and synthetic matrices [36]. Natural matrices, such as BCs, platelet-rich plasma (PRP), platelet-rich fibrin (PRF), collagen, and hyaluronic acid, are either autologous or naturally derived. Bus, commonly used in cell homing due to their simplicity, allow cell surface integrins to adhere to the fibrous component and provide growth factors necessary for tissue regeneration [35-36]. However, their use has limitations, such as challenging extraction, suboptimal mechanical properties, and uncontrolled biodegradability [33]. Platelet-derived scaffolds, such as PRP and PRF, offer benefits including ease of preparation and a rich supply of bioactive molecules, essential for cell homing [33,35-36]. These scaffolds, however, require special equipment for blood collection and lack precise control over growth factor types and concentrations, as well as mechanical strength and degradation timing. Naturally derived scaffolds, including polysaccharides and proteins like alginate, chitosan, collagen, and fibrin, are beneficial for their biocompatibility and integration with tissue regeneration processes in cell homing [35-36]

Indications and contraindications

Regenerative endodontic therapy via cell homing is indicated in patients who have necrotic permanent teeth with incomplete root formation, with or without periradicular lesions. It is suitable for structurally sound teeth that do not require a post or core for final restoration and for patients who are cooperative with no known drug allergies. On the contrary, regenerative endodontic therapy is not indicated for teeth that have been replanted after avulsion or for grossly carious teeth with extensive loss of tooth structure that requires a post and core. It is also inappropriate for teeth with inadequate access or isolation and for teeth that have combined endodontic and periodontal lesions [37].

Case selection

Age

Research has shown that younger patients, especially those between the ages of nine to 13 years, are better candidates for regeneration through cell homing compared to older patients above 14 years [38]. This is attributed to the fact that younger patients possess a greater capacity for healing and regeneration, and this capacity tends to decrease with age [39]. Another important age-related factor is the stage of root development. A larger apex diameter allows for more tissue ingrowth into the canal space, which will provide a rich source of MSCs from the apical stem cells. However, regenerative procedures are not recommended for deciduous teeth, as the process of inducing a BC may disrupt the eruption pattern of the succeeding permanent tooth [40].

Root Morphology

Root morphology plays a very important role in the success of regenerative procedures. However, studies on apical diameter have revealed conflicting results. Some studies indicated that smaller apical diameters, ranging between 0.24 mm to 0.53 mm, produced better outcomes with regenerative endodontic procedures (REPs) [41]. By contrast, another study documented an increase in tissue ingrowth in the root canals with a rise in apical diameter [42]. Recently, REPs were performed on mature teeth with closed apices and pulp and periapical disease; the periapical lesions resolved and vitality testing showed a positive response [43].

Medical History

Before starting the cell homing process, it is of utmost importance to take a detailed medical history from the patient. It is crucial to inquire about any relevant medical conditions that might affect the outcome of the treatment. For example, patients with hypertension may experience more bleeding than usual. Those with a history of cardiovascular disease might be on anticoagulant drugs, and it is important to temporarily discontinue these medications. Not only that, patients with a history of coronary artery and cerebrovascular diseases are not ideal candidates for regenerative endodontics. Patients with diabetes are more prone to infections, and infection control can be challenging in patients who are on steroids or hormone therapy. Special care should be exercised in managing patients with mental disabilities and anxiety and those who are unwilling or unable to commit to long-term follow-up [37].

Treatment protocol

Before starting with the actual REP procedure, it is necessary to provide the following information to the patient’s guardian or parents: the present status of the affected tooth, the procedure the endodontist is planning to perform, the duration and frequency of the treatment and follow-up visits, the cooperation required from the patient, the result of the treatment, both positive and negative potential outcomes, alternative treatment options, and the cost of the treatment. Once the patient’s guardian has a thorough understanding of the procedure, the next step is to obtain informed consent [37,44].

This is followed by a detailed intra-oral examination, pulp vitality testing, and then radiographic assessment to study the root morphology and to rule out the presence of any periapical lesion or periodontal conditions [37,44].

Even though there are some variations in the treatment protocol designed by the American Association of Endodontists (AAE) and the European Society of Endodontology (ESE), the basic requirements remain the same. They include minimal instrumentation, proper chemical disinfection, use of adequate intracanal medicaments, creation of an optimum BC, placement of a scaffold over the induced BC, and the appropriate placement of bioceramic material, followed by an effective coronal seal with glass ionomer cement (GIC) and composite [37,44]. The treatment procedure of REP is divided into two visits.

In the first visit, superficial disinfection of the oral cavity is done with 2% chlorhexidine mouthwash. Local anesthesia is then induced, and the use of vasoconstrictors with the anesthetic agent can be avoided. The tooth to be treated is isolated with a rubber dam. Following isolation, the carious or infected part of the tooth is removed, and access cavity preparation is done under a microscope. Once the access cavity is prepared, the working length is determined with the help of a radiograph. The file should be positioned 1 mm from the apex. Minimal instrumentation of the canal is performed with large-size reamers, as the diameter of the open apex of the tooth is usually larger than the biggest file. This step is performed by gently brushing the file against the canal wall, and care is taken to remove only infected dentin without vigorous instrumentation. Irrigation is done with 1.5-3% sodium hypochlorite and saline. The needle should be 2 mm short of the root apex to avoid trauma to the periapical tissues. This step is followed by irrigation with EDTA. Next, the cavity is dried with paper points, and intracanal medicament is placed below the cementoenamel junction. The AAE suggests the use of Ca(OH)2 or antibiotic dressing, whereas the ESE recommends only Ca(OH)2. The access cavity is then closed with a temporary restoration, which should be at least 3-4 mm thick [37,44].

The second visit is scheduled after one to four weeks per AAE guidelines or two to four weeks per ESE suggestions. During the second visit, the patient is evaluated for any signs and symptoms of persistent infection. If there is an infection, all the procedures of the first appointment are repeated, and another recall visit is planned after one week. Once the infection has subsided, the endodontist can proceed with the procedures of the second appointment. Local anesthesia is again induced with a 3% mepivacaine anesthetic agent without a vasoconstrictor agent (epinephrine). A rubber dam is placed, and superficial disinfection is done with 2% chlorhexidine or 2% povidone-iodine [37,44]. The temporary restoration is removed, and the canal is irrigated with 17% EDTA. The AAE does not have specific recommendations for the time and volume of irrigation with EDTA for this procedure, while the ESE recommends 20 ml of EDTA for five minutes followed by 5 ml of saline irrigation. If needed for effective medicament removal, ultrasonic activation can be performed. Once the medicament is removed, the canal is dried with absorbent paper points. After the canal is completely dry, bleeding is induced by rotating a larger-sized K file 2 mm below the apex. This is done until the entire canal is filled with a BC below the cementoenamel junction. A waiting period of 15 minutes is observed to allow for clot formation. Once the BC is formed, a resorbable matrix, such as CollaPlug™, CollaCoat™, or CollaTape™, is placed over the BC. The diameter of the resorbable matrix is kept slightly larger than the coronal part of the root canal. The matrix is covered with tricalcium silicate biomaterial like MTA or fast-set putty, and this material is placed 2 mm below the CEJ. Finally, a 3-4 mm layer of light-cured glass ionomer cement is placed on top of the biomaterial, followed by a permanent restoration of the reinforced composite [37,44].

The follow-up visits are usually scheduled at three months, six months, and 12 months. This is followed by a yearly visit for the next five years. Each follow-up visit includes clinical assessment and radiographic examination [37,44].

Clinical considerations

Minimal Mechanical Instrumentation

A major challenge in treating permanent teeth with open apices is that the diameter of the apex often exceeds the size of the largest file. In addition, the dentin lining the walls of root canals is very thin. Therefore, utmost care must be taken to not over-instrument the canal walls; this will help preserve the thickness of the dentin. At the same time, mechanical instrumentation is vital. Without it, bacteria may remain embedded in the dentinal tubules and increase the chances of re-infection [45]. Minimal mechanical instrumentation and the use of larger-sized K files for the effective removal of infected dentinal wall without disrupting the thickness are suggested.

Chemical Disinfection

The most important roles played by chemical disinfectants in REP are the dissolution of tissue, elimination of endodontic infection, and acceleration of the release of growth factors from the dentinal walls. The most commonly used chemical disinfectants in REP are sodium hypochlorite and 17% EDTA. The optimal concentration for NaOCl is still uncertain, but studies have demonstrated a significantly lower survival rate of stem cells with 6% NaOCl [46]. Therefore, lower concentrations are recommended. In addition, 17% EDTA positively impacts the initiation, proliferation, and differentiation of stem cells along with the release of growth factors from dentin [47]. Both the AAE and ESE recommend 1.5-3% NaOCl (20 mL/canal for five minutes), followed by 17% EDTA (20 mL/canal for five minutes) in the first appointment and only 17% EDTA without NaOCl in the second appointment. Importantly, care should be taken to not extrude the irrigants into the periapical tissue to minimize cytotoxicity to periapical stem cells. To be specific, the needle should be placed 1-2 mm from the apex. For enhanced effects, irrigants can be combined with lasers, ultrasonic irrigation, and negative pressure irrigation [47].

Intracanal Medication

The recommended intracanal medicaments are triple antibiotic paste (TAP), double antibiotic paste (DAP), and calcium hydroxide (Ca(OH)2) [48]. TAP contains ciprofloxacin, metronidazole, and minocycline in a 1:1:1 concentration. This paste has been reported to have a success rate of up to 80% in REP, but its effect on the viability of stem cells is detrimental. Therefore, the AAE recommends 1-5 mg/mL of TAP. However, TAP can discolor the tooth due to minocycline and should always be placed below the CEJ [48]. To avoid this discoloration, a dentin bonding agent can be applied before placing the intracanal medicament. As an alternative to TAP, DAP can be used, in which minocycline is either completely omitted or replaced with clindamycin or cefaclor [48-49]. Although Ca(OH)2 has comparatively less antibiotic potency than TAP or DAP, its other qualities make it superior. Studies have shown that in the presence of calcium hydroxide, there is greater survival and proliferation of stem cells, as well as no discoloration, promotion of growth factor release from dentinal walls, and easier removal from the canals [49]. The chances of tooth fracture are also drastically reduced since Ca(OH)2 is used for a comparatively shorter period of one to four weeks. Because of these qualities, ESE recommends the use of Ca(OH)2 for the successful outcome of REPs. Recent advancements have explored the use of propolis as an antibiotic paste, although its efficacy in humans is yet to be tested [50]. Another study investigated the use of nanofibers for the intracanal delivery of medications [51].

Formation of a BC

After the canals are successfully disinfected, the periapical tissue of the tooth is lacerated to induce bleeding and create a BC that acts as a scaffold. This scaffold promotes growth factors and stem cell migration from the apical region to the canal. It is most commonly used in the case of non-vital permanent immature teeth. The clinician may at times experience inadequate intracanal BC formation, which can be a challenge for a successful REP [48]. Insufficient clot formation could occur due to the use of vasoconstrictors along with the anesthetic agent, rapid resolution of inflammation following intracanal medicament, or the destruction of periapical tissues from over-instrumentation [52]. Therefore, both AAE and ESE suggest using an anesthetic agent without vasoconstrictors in the second appointment. Another alternative to induce intracanal bleeding is IV or IA introduction of PRP, PRF, or controlled growth factors (CGF) [53]. Studies evaluating the potency of BC induction along with IV or IA injection of scaffolds did not observe any significant variation in outcomes [54].

Achieving a Proper Coronal Crown Seal

As mentioned in the treatment protocol, proper coronal seal after the induction of a BC plays a vital role in the successful outcome of REP. Once the BC is induced, it is covered with a matrix, and tricalcium silicate biomaterial is placed on it. Above the MTA, a 3-4 mm layer of glass ionomer cement is placed, which is again covered with a bond-reinforced composite restoration [37,44].

Sequelae of cell homing

Radiological Outcomes

A tooth typically exhibits five types of responses after undergoing regenerative endodontic treatment. The type 1 radiological outcome indicates an increase in canal wall thickening along with continued root development. In type 2, the root apex appears blunt and closed, with less significant development. Type 3 shows continued maturation of the root, but the apical foramen remains open. Type 4 is characterized by significant calcification in the canal space, which may obliterate the entire canal. In type 5, a hard tissue barrier may have formed between the coronal MTA and the root apex [55].

Histological Outcomes

Healing by repair: The histological sequence of healing in regenerative endodontics is a multifaceted process. It involves the formation of various tissue types, with dentin-like, cementum-like, and bone-like tissues developing to different extents. Dentin-like hard tissue is less commonly formed, while cementum-like hard tissues are frequently observed on the dentinal walls. Bone-like tissue formation is also present but not as prevalent as cementum-like tissue. Scaffolds are integral to this process, influencing the outcome of tissue regeneration. BC as a scaffold leads to the formation of various tissue types, including dentin-like, cementum-like, and bone-like tissues. The addition of other materials to BC or the use of alternative scaffolds results in varied tissue formation. The vitality and structure of the newly formed tissues are notable. Vital tissues, mainly consisting of connective tissue infiltrated with fibroblast-like cells and blood vessels, are seen in the canal space with different scaffolds. Some studies report the formation of periodontal ligament (PDL)-like tissues and, in fewer cases, pulp-like tissue. In essence, regenerative endodontics encompasses the formation of diverse tissue types, with scaffolds playing a crucial role in this process. This sequence reflects the complex biological mechanisms involved in dental tissue repair and regeneration [56].

Response to Pulp Vitality Testing

A multilevel approach was designed to evaluate the success of REPs. Three stakeholders were involved: patients and their parents (patient-centered), clinicians (clinician-centered), and scientists (scientist-centered) [57]. Patient-centered outcomes are considered the top priority. They include the resolution of infection, which presents as reduced swelling, relief from pain, and healed draining sinus; the survival of the teeth; and acceptable esthetics. From a clinician’s perspective, a successful REP outcome is measured in terms of radiographic signs of healing of the periapical lesion and root development, along with a positive response to pulp vitality tests. For a scientist, the ultimate goal is the histologic evidence of complete regeneration of pulp tissue in treated teeth. However, among all three stakeholders, the patient’s experience is given the most importance [57].

Success Criteria for Regenerative Endodontics Through Cell Homing

The AAE has established three sets of goals as success criteria for regenerative endodontics, which are classified as primary, secondary, and tertiary goals. The primary goal is to eliminate the symptoms and achieve bony healing as evaluated through radiographic imaging [37]. A study was conducted on 50 immature permanent teeth with necrotic pulp and periradicular pathosis treated with the cell homing technique; after evaluation using cone beam computed tomography (CBCT), it was found that only two teeth showed chronic periapical abscess, indicating a 94.6% success rate for regenerative endodontics with the cell homing technique [58]. Moreover, a systematic review documented high probabilities of success in resolving the signs and symptoms of infection and periapical healing, at 91% and 94%, respectively [59]. The secondary goal is to increase root wall thickness and/or root length; this goal is desirable but not essential. Another CBCT study on five infected immature permanent teeth treated with the cell homing technique evaluated periapical bone healing, root development, and pulp vitality. The results demonstrated an increase in root hard tissue volume and root length, but these were five and three times less, respectively, compared to contralateral teeth [60]. The tertiary goal is to elicit a positive response to vitality testing and therefore confirm the regeneration of vital pulp tissue. A positive response to a vitality test suggests innervated and live pulp tissue. However, the response depends entirely on the patient’s perception of sensation, which could be misleading. A clinical trial documented pulp vitality testing in 15 mature necrotic teeth treated with cell homing and PRF membrane; six out of 15 teeth showed a positive response to vitality test and had intensities of over 40 on a scale of 80 [61].

According to the ESE, the success criteria for REPs include the absence of inflammatory signs, healing of any pre-existing periapical bony lesion, increased root length and wall thickness, lack of external inflammatory resorption, a positive response to pulp vitality testing, new PDL formation along the inner wall of the root canal as seen on a radiograph, and no tooth discoloration [44].

Complications and limitations

During REPs, a clinician may encounter complications, such as the perception of pain by the patient, tooth discoloration due to the use of intracanal medicament, BCs, and in extreme cases, intra-canal calcifications. The clinician should be prepared to prevent these issues and manage them, should they arise.

Pain

A patient may experience pain due to excessive instrumentation of the canal, which could potentially traumatize the periapical tissue [44,48]. This often occurs due to improper determination of the working length, resulting in the overextension of files and irrigating needles beyond the apex. Instrumentation to induce the BC, essential in REP, should be performed during the second appointment [48]. If over-instrumentation occurs in the first appointment, it may lead to inadequate BC formation in the second visit and compromise the procedure’s outcome. Pain may also arise if there is insufficient anesthetic effect before beginning treatment. Therefore, it is recommended to use an anesthetic agent with good permeability to control pain during the procedure. Another cause of pain is the improper removal of infected dentin due to inadequate isolation, which could lead to residual infection or reinfection of the canal between appointments. This pain can be prevented or controlled by using 3% mepivacaine [44]. To eliminate the risk of intra-canal infection, copious irrigation with EDTA and NaOCl is recommended, followed by the use of adequate intracanal medicament [47]. Furthermore, it is crucial to prevent microleakage between appointments by tightly sealing the cavity. If the patient experiences pain between appointments, disinfection of the root canal should be repeated until symptoms subside. However, if the pain continues, alternative treatment plans, such as apexification or extraction, should be considered after informing the patient of the situation.

Tooth Discoloration

Discoloration is a common outcome of REP, with the literature stating that nearly 40% of patients experienced discoloration after REP [62]. Therefore, the possibility of discoloration and its management should be discussed with the patient beforehand. While discoloration may be acceptable in the posterior teeth, it can impact aesthetics and quality of life if it occurs in the anterior teeth. Discoloration can occur at any stage of treatment. During the first appointment, if TAP is used as an intracanal medicament, minocycline can chelate calcium ions, deposit them up to 350 µm into dentinal walls, and stain the teeth [63]. Even after vigorous irrigation, a significant portion of the medication remains in the canals. To avoid staining, the medicament should be placed below the cementoenamel junction. Alternatively, DAP can be used instead of TAP. Furthermore, the induction of a BC in the second stage of treatment can also cause staining [64]. Components of blood, like hemoglobin, may incorporate into the dentin and alter the tooth’s refractive index. In addition, sealing materials can cause discoloration due to their chemical compositions [65]. Discoloration can be treated with internal bleaching, or in extreme cases, the stains can be concealed with composite.

Intra-canal Calcification

Intra-canal calcification, identified as revascularization-associated intra-canal calcification (RAIC), can manifest as a calcific barrier (CB) in the midroot region or cause complete obliteration of the root canal [66]. Research has suggested that PDLSCs from the periapical area and BMSCs from the alveolar bone migrate to the canal along with the induced BC. As these stem cells retain their innate potential to form cementum and bone, they can lead to canal obliteration. Initially, it was assumed that only the BC was responsible for this, but calcifications have also been observed in patients who had IV or IA injections [66]. The materials used to cover the scaffold facilitate the initiation and differentiation of MSCs. This process promotes odontogenesis and creates an alkaline environment in the root canal that stimulates tumor growth factors, thus enhancing the formation of mineralized tissue. However, calcified tissue within the canal typically does not cause problems unless the tooth becomes symptomatic. In cases of reinfection, prompt intervention with microendodontic surgery is indicated, and in extreme cases where the infection is beyond treatment, tooth extraction may be necessary [41,66].

Future scope

To date, enhancing the homing abilities of stem cells for therapeutic applications remains the primary challenge of cell homing. This is largely due to the low expression of homing molecules. Consequently, various strategies have been proposed to augment stem cell homing, such as non-systemic targeted delivery, which involves the direct delivery of MSCs into the specific target region. In addition, magnetic guidance has been found to efficiently direct MSCs to target tissues. The surface engineering of stem cells, such as coating their surface with hyaluronic acid, has also been advocated to promote overexpression. Another similar approach is to modify the target tissue to make it more attractive to stem cells. Lastly, genetic modifications of MSCs, which can be achieved through overexpression, are seen as a method of enhancing homing.

## Conclusions

Within the field of regenerative endodontics, recent developments emphasize the crucial role of cellular homing, the use of scaffolds, and the impact of growth factors in the regeneration of dental pulp tissue. This area of study acknowledges the intricacies involved in mimicking the pulp-dentin structure, highlighting the significance of dental stem cells and their ability to differentiate. In addition, it explores various clinical methodologies and the potential challenges associated with these regenerative procedures, signifying a notable advancement in dental tissue repair and offering promising prospects for future dental treatments.
